# Neural Representation of Overlapping Path Segments and Reward Acquisitions in the Monkey Hippocampus

**DOI:** 10.3389/fnsys.2019.00048

**Published:** 2019-09-12

**Authors:** Rafael Vieira Bretas, Jumpei Matsumoto, Hiroshi Nishimaru, Yusaku Takamura, Etsuro Hori, Taketoshi Ono, Hisao Nishijo

**Affiliations:** ^1^System Emotional Science, Graduate School of Medicine and Pharmaceutical University, University of Toyama, Toyama, Japan; ^2^Symbolic Cognitive Development, Center for Biosystems Dynamics Research, RIKEN, Kobe, Japan

**Keywords:** hippocampus, place cells, navigation, overlapping, disambiguation, neural differentiation

## Abstract

Disambiguation of overlapping events is thought to be the hallmark of episodic memory. Recent rodent studies have reported that when navigating overlapping path segments in the different routes place cell activity in the same overlapping path segments were remapped according to different goal locations in different routes. However, it is unknown how hippocampal neurons disambiguate reward delivery in overlapping path segments in different routes. In the present study, we recorded monkey hippocampal neurons during performance of three virtual navigation (VN) tasks in which a monkey alternately navigated two different routes that included overlapping path segments (common central hallway) and acquired rewards in the same locations in overlapping path segments by manipulating a joystick. The results indicated that out of 106 hippocampal neurons, 57 displayed place-related activity (place-related neurons), and 18 neurons showed route-dependent activity in the overlapping path segments, consistent with a hippocampal role in the disambiguation of overlapping path segments. Moreover, 75 neurons showed neural correlates to reward delivery (reward-related neurons), whereas 56 of these 75 reward-related neurons showed route-dependent reward-related activity in the overlapping path segments. The ensemble activity of reward-related neurons represented reward delivery, locations, and routes in the overlapping path segments. In addition, ensemble activity patterns of hippocampal neurons more distinctly represented overlapping path segments than non-overlapping path segments. The present results provide neurophysiological evidence of disambiguation in the monkey hippocampus, consistent with a hippocampal role in episodic memory, and support a recent computational model of “neural differentiation,” in which overlapping items are better represented by repeated retrieval with competitive learning.

## Introduction

The hippocampal formation (HF) has been implicated in human episodic memory and spatial navigation (Scoville and Milner, 1957; O’Keefe and Nadel, 1978; Squire and Zola-Morgan, 1991; Tulving and Markowitsch, 1998; Burgess et al., 2002). Consistent with these roles of the HF, neurophysiological studies have also reported that HF place cells code subject’s own position in a specific place of the environment that the rodents navigate (O’Keefe and Dostrovsky, 1971; McNaughton et al., 1983; Muller and Kubie, 1987; Eichenbaum et al., 1990). In monkeys, the activity of place-related neurons in the HF increased when the subjects navigated a particular location in a virtual or real environmental space (Ono et al., 1993; Matsumura et al., 1999; Ludvig et al., 2004; Hori et al., 2005; Furuya et al., 2014; Wirth et al., 2017; Hazama and Tamura, 2019). This has also been demonstrated in the human HF (Ekstrom et al., 2003; Miller et al., 2013). It has been proposed that these place cells play an important role in episodic memory (O’Keefe and Nadel, 1978; Eichenbaum, 2017).

The ability to distinguish overlapping items in time and space is critical to episodic memory. Human fMRI studies have reported that the HF encodes distinctly the same path segments in different routes (Brown et al., 2010; Chanales et al., 2017). In rodent studies, animals navigated different routes that shared a common path segment, and some HF place cells fired differently in this segment depending on the routes (Frank et al., 2000; Wood et al., 2000; Ferbinteanu and Shapiro, 2003; Dayawansa et al., 2006; Ainge et al., 2007, 2012; Grieves et al., 2016). That is, the neurons differentially fired in the same position depending on where the animal had come from or where it was going (route-dependent activity). These results suggested that the HF plays a role in prospective and retrospective coding in episodic memory, in which a sequence of specific behaviors is coded in association with spatial information (Catanese et al., 2014). However, route-dependent neuronal activity in non-human primates remains unknown.

Neuronal responses to rewards are another important factor of episodic memory. A human fMRI study reported that reward values associated with items affected the encoding of those items in the HF (Kuhl et al., 2010). The HF receives reward-related signals from dopaminergic, cholinergic, and amygdalar neurons (Lisman and Grace, 2005; Terada et al., 2013; Teles-Grilo Ruivo et al., 2017). Furthermore, the activity of rodent and monkey HF neurons changed in response to reward delivery or locations associated with reward (Rolls and Xiang, 2005; Ho et al., 2008, 2011; Xia et al., 2017). A recent study using rodents reported that some HF neurons specialized for encoding reward location are active not only in one environment but also across multiple environments (Gauthier and Tank, 2018). However, it is unknown whether reward-related neurons are active in multiple environments in primates. Furthermore, it is also unknown how HF neurons disambiguate reward delivery in overlapping situations in both rodents and primates.

Thus, two types of HF neurons, place cells coding subject’s own positions and reward-related neurons (goal-directed cells) coding locations of goals, are supposed to play an important role in episodic memory as well as navigation to a goal in rodent and bat HF (Burgess and O’Keefe, 1996; Poucet and Hok, 2017; Sarel et al., 2017). However, it is unknown how these HF neurons contribute to disambiguation of routes and reward delivery in primates.

We hypothesized that both of these types of HF neurons were involved in disambiguation of routes and reward delivery in monkeys, and that environments surrounding a maze could affect responsiveness of reward-related neurons (i.e., different reward-related neurons would respond in different environments) since a previous study reported that monkey HF neurons were sensitive to the surrounding environment of the maze (Hori et al., 2005). In the present study, the monkeys navigated along a figure 8-shaped track to acquire rewards in a virtual environment, and we analyzed the HF neuronal activity while they passed the overlapping segments and received the reward. We examined whether population activity of place-related and reward-related HF neurons in the overlapping path segments disambiguates overlapping items (navigation and reward delivery in the same path segment but in different routes), and as well as whether reward-related activity across different environmental settings could differentiate multiple environments.

## Materials and Methods

### Animals

Two male adult Japanese monkey (Macaca fuscata), weighing 9.0 (monkey A), and 10.5 (monkey B) kg, respectively, were used in the current experiment. The monkeys were housed individually in the home cage and supplied with monkey rations *ad libitum* and daily fruits or vegetables. Environmental enrichment, in the form of toys, was provided daily. Although the animals were deprived of water in the home cage, they were able to receive liquid reward during the experimental session. Supplemental water and vegetables were given after each day’s session. To assess the monkey’s health, their weight was routinely monitored. The experiment was conducted in strict compliance with the United States Public Health Service Policy on Human Care and Use of Laboratory Animals, the National Institutes of Health Guide for the Care and Use of Laboratory Animals, and the Guidelines for the Care and Use of Laboratory Animals at the University of Toyama. The experimental protocol was approved by the ethical committee for animal experiments in the University of Toyama.

### Experimental Apparatus

**FIGURE 1:**
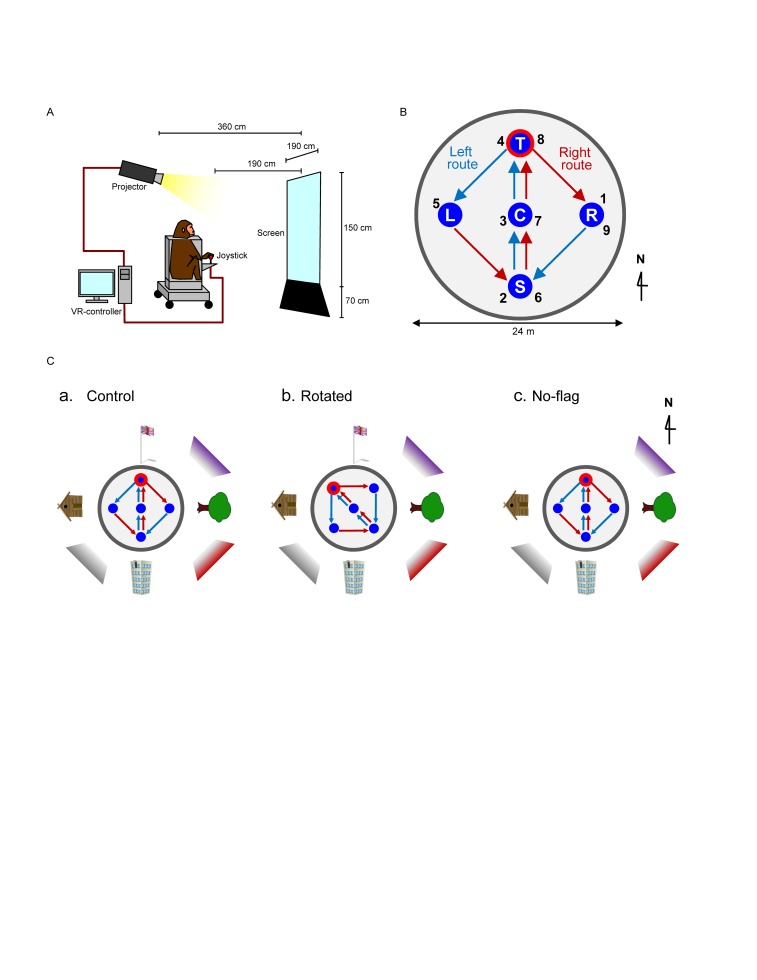
Schematic illustration of virtual navigation (VN) in monkeys. **(A)** Experimental set up. **(B)** Movable area. In the task, the subject was required to navigate the movable area in a specific sequence. In the left route (blue arrows), the monkey was required to navigate, and visit the reward areas in the following order: location sequence numbers 1–5. In the right route (red arrows), the monkey was required to navigate, and visit the reward areas in the following order: location sequence numbers 5–9. The common central hallway (location sequence numbers 2–4 and 6–8) was overlapped by the two routes. **(C)** Spatial arrangement of the three VN tasks used in the current study.

During the recording session, the animal was placed on a restraining chair and had its head painlessly fixed with an acrylic U-shaped frame that was surgically implanted into the monkey’s skull, which worked as a movement restrainer (Ono et al., 1993; Matsumura et al., 1999; Hori et al., 2005; Furuya et al., 2014). The chair consisted of an acrylic box with wheels, in which the monkey could be transferred from its home cage to the experimental room. Inside this box, the monkey could sit comfortably to perform the task. An infrared charge-coupled device (CCD) camera for eye-movement monitoring was firmly attached to the chair with a steel rod. During recording sessions, the monkey’s eye position was monitored with 33 ms time resolution using an eye-monitoring system (Matsuda, 1996). In the experimental room, the chair was positioned 2.6 m away from a wide projector panel, which was 1.5 m high × 1.9 m wide, displaying 3D polarized images projected by an LCD projector located behind and above the monkey (Figure 1A). The animal was trained to perform the task by looking at the screen using polarized lenses, attached to the outer part of the chair, i.e., as if the monkey was wearing 3D polarized glasses. During the task, the room lights were turned off and the animal had no view of the experimenter.

A joystick was attached to the front wall of the chair. The animal could acquire a liquid reward (i.e., sports drink) by manipulating the joystick through a window in the chair. Liquid delivery was controlled using an electromagnetic valve connected to a tube projecting through the rear side of the monkey’s chair. The monkey could not reach any object other than the joystick.

### Behavioral Paradigms

The animals were first trained to perform a control virtual navigation (VN) task, where they were required to navigate in a 3D environment by manipulating the joystick (Hori et al., 2005; Furuya et al., 2014). For this task, a large 3D open-field space with a 180-m diameter was created using a 3D software (EON Studio ver. 2.5.2, EON Reality, United States) (Figure 1B). However, the monkey could move only inside a limited 24-m diameter space located in the center of the open-field, surrounded by a wall (with a height of 0.5 m). This central part of the virtual space will be henceforth referred to as the mobility area. The open field contained five reward areas (with a diameter of 2.8 m) that were placed in the center and apex of a diamond shape within the mobility area (S, C, T, L, and R in Figure 1B). Extra-maze cues (i.e., one tree, one hut, one building, and one flag) were located 2.0 m away from the wall outside the mobility area. Another extra-maze reference points (i.e., three posters) were located on the background, serving as the distal cues.

In this task, the animal was required to track the reward areas in a specific order; (1) R → S → C → T → L (location sequence from No. 1 to 5; left route), and (2) L → S → C → T → R (location sequence from No. 5 to 9; right route) (Figure 1B). The animal received a liquid reward immediately after entering each reward area in the correct sequence; the monkey could obtain a total of 8 rewards in each trial. No delay time before reward delivery was imposed in each reward area. Thus, the left and right routes shared the same path segment (common central hallway). The movements on the common central hallway were associated with right and left turn at T-reward area, respectively. Each HF neuron was tested with at least 7 trials.

One monkey (monkey A) was trained only with a control environment (a control VN task). Spatial arrangement of the distal cues in the control VN task is shown in Figure 1Ca. To investigate reward-related activity in multiple environments (see section “Introduction”), another monkey (monkey B) was trained not only in the control VN task but also in two different environments (rotated and no-flag VN tasks). In the rotated VN task, the reward locations in the movable area and two routes were rotated by 45° in the counter-clockwise direction (Figure 1Cb). In the no-flag VN task, the spatial arrangement of the distal cues was the same as that in the control VN task except that the flag was eliminated (Figure 1Cc). The intra-maze cues and routes were identical in all these different virtual spaces.

### Training

Initially the monkeys were trained on a pointer translocation task in which it learned to operate the joystick (Furuya et al., 2014). First, they had to move a large pointer to two different reward areas with a large size on the screen. Initially, only movements in one dimension were allowed, under a virtual guide path making impossible for the cursor to leave the horizontal or vertical axis. As they became more skillful, the virtual guide was removed, the size of the reward areas was gradually decreased and locations of the reward areas moved apart from each other until the animal developed the ability to control the joystick on every axis. It took 4 months for the monkeys to learn to move the joystick freely in all directions without the cursor movement limitation. When the monkeys could perform the cursor translocation task with a criterion of 99% correct responses, they were moved to the next level and training for the VN tasks began.

For the VN training, the animal had to get used to the VN environment as well. In the first step of training the subject manipulated a monkey avatar on the screen from a top down view. Each day the camera angle and distance from the avatar was decreased. At the end of the training the subject performed from a first-person view in a real-world like perspective. The monkey initially manipulated the joystick under physical limitations, made with a metal plate below the joystick, which allowed three movements simultaneously to the left, right and front only, so the animal could learn that he must be facing the reward area to acquire the reward. By moving the joystick to the front, the monkey could move forward in the virtual space. By moving the joystick to the right and left, the monkey could turn clockwise and anti-clockwise in the virtual space, respectively. Both monkeys were initially trained in the control VN task, and then monkey B was further trained in the rotated and no-flag VN tasks.

### Surgery

After the completion of the training period (approximately 1 year), the animal was implanted with a head movement restrainer (U-shaped acrylic frame) on the skull (Hori et al., 2005; Furuya et al., 2014). The surgical procedure was conducted under aseptic conditions. The animal was anesthetized with a combination of medetomidine hydrochloride (0.5 mg/kg, i.m.) and ketamine hydrochloride (5.0 mg/kg, i.m.). The frame was anchored with dental acrylic to tungsten bolts inserted in the skull. During the surgery, heart, respiratory functions, and rectal temperature were monitored (LifeScope14, Nihon Kohden, Tokyo, Japan). A thermal blanket was used to maintain the body temperature at 36 ± 0.5°C. Antibiotics were administered topically and systemically for 1 week to prevent infection. Two weeks after the surgery, training was resumed with the subject’s head fixed to the stereotaxic apparatus. Performance criterion (95% correct ratio) was once again attained within 2 weeks. A brain MRI was acquired and stereotaxic coordinates of the target area were checked against the stereotaxic atlas (Kusama and Mabuchi, 1970). Finally, the subject was again anesthetized with ketamine hydrochloride (0.5 mg/kg, i.m.) and a hole was opened in the animal’s skull above the target area, so that the electrode could be inserted in the recording sessions.

### Recording Procedures and Data Acquisition

After the monkey was placed in the monkey chair, a quartz insulated platinum tetrode (Thomas Recording GmbH, Giessen, Germany; *Z* = 0.6–1.0 MΩ at 1000 Hz) was stereotaxically inserted stepwise with a pulse motor-driven manipulator (SM-21S, Narishige, Tokyo, Japan) into various parts of the HF. In monkey A, neuronal activity was recorded from the left HF while the monkey performed the control VN task. In monkey B, neuronal activity was recorded from the right HF while the monkey performed the three VN tasks. Data on the analog signals of neuronal activities, triggers for the liquid reward, X-Y coordinates of the monkey in the virtual space, joystick positions, and eye position were digitized and stored on a computer via a Multichannel Acquisition Processor system (Plexon Inc., Dallas, TX, United States). The amplified neuronal signals were digitized at a 40-kHz sampling rate; 800-μs waveforms that crossed an experimenter-defined threshold were stored on a computer hard disk for offline spike sorting.

### Unit Identification

The digitized waveforms of the isolated units were superimposed to check for invariability during the recording sessions. The data were then transferred to the analysis software NeuroExplorer (Nex Technologies, Littleton, MA, United States). Recorded waveforms were projected to a principal component subspace using NDManager (Hazan et al., 2006)^1^ and semi-automatically sorted into single units using KlustaKwik (Harris et al., 2000)^2^ and Kluster (Hazan et al., 2006; see text footnote 1) as outlined by previous studies (e.g., Maingret et al., 2016; Patrono et al., 2017). Each cluster of neuronal spikes was then assessed manually to ensure that the cluster boundaries were well separated and that the waveform shapes were consistent with action potentials. Further, an autocorrelogram was constructed for each isolated cluster. An absolute refractory period of at least 1.0 ms was used to exclude suspected multiple units.

### Neural Correlates to Space

For the analysis of place fields, the mobility area in the VN tasks was divided into 30 × 30 pixels grids. The mean firing rate for each pixel was defined as the average number of spikes per second for all visits to that pixel during VN. Then, a whole task mean firing rate (M) for each VN task was calculated by averaging the mean firing rate during the whole task duration. Finally, the firing rate was smoothed using a Gaussian function according to a 3-pixel radius. If the subject did not visit the same pixel for at least 300 ms during the task, those pixel data were not considered for place field analysis.

Place fields in the VN tasks, which were defined as the pixels in which the activities of the HF neurons increased, were identified based on the mean firing rates (Furuya et al., 2014). Only place fields that had at least one pixel with a mean firing rate exceeding twice the mean firing rates and one adjacent pixel with a mean firing rate exceeding 1.5-times the mean firing rates were analyzed separately in each route in each VN task. The place fields could be expanded through any edge shared by two pixels meeting the criterion (>1.5-times the mean firing rates). If one or more neighboring pixels satisfied the criterion, the field was expanded to include those pixel(s). Each added pixel was then tested for the presence of a neighboring pixel that met the criterion. When no neighboring pixel satisfied the criterion, the limit of the field was identified. The minimum size for a place field was set at 9 pixels with a minimum of three visits during the task. Place-related neurons in the VN tasks were defined as neurons that displayed the place field(s), as noted above, in the either left or right route, in at least one of the three VN tasks. The definition of place field in reference to the total mean firing rates was based on previous studies (Muller et al., 1987; Kobayashi et al., 1997; Matsumura et al., 1999; Harvey et al., 2009). The monkey’s trajectory was divided into two routes as follows: the left and right routes. To make sure the place field(s) located in the transition between the two routes were detected, there was a 1-s overlap between the left and right routes: the initial 1-s data of the right route included the last 1-s of the left route before entering the left reward area, while the initial 1-s data of the left route included the last 1-s of the right route before entering the right reward area. Place fields were separately analyzed using data in each route. The firing rate maps of either the left or right route in the control VN task were used as the control. The firing rate maps in the other tasks were also constructed separately for the left and right routes.

To analyze effects of different routes on place-related activity in the same path segment, the common central hallway between the two reward locations (C and T) was divided into three zones (i.e., zones 1, 2, and 3). Neural activity in the common central hallway was analyzed using a two-way analysis of variance (ANOVA) with zone and route as factors. Neurons that displayed a significant main effect of route (*p* < 0.05) and/or a significant interaction between zone and route (*p* < 0.05) were defined as route-dependent neurons.

### Neural Correlates to Reward Delivery

Mean firing rates around reward delivery were analyzed using one-way and three-way ANOVAs to examine whether reward influenced the activity of the HF neurons. A one-way ANOVA using peri-event histograms constructed during a period of 4 s (2 s before and 2 s after reward delivery) in successive 1-s bins in each reward area, was used to estimate the neural responses to rewards. Reward-related neurons were defined as such if they showed a significant main effect at least at one of the reward areas in either the left or right route.

To examine the effects of routes on responses to reward delivery in the common central hallway, the firing rates were analyzed using a three-way ANOVA with reward area (T vs. C vs. S), period (two 1-s periods before and after reward delivery), and route (left vs. right route) as factors. The responses to rewards were considered to be modulated by routes (route-dependent reward-related neurons) if a significant main effect of route and/or significant interactions between reward area and route, period and route, or reward area, route, and period were observed (*p* < 0.05). For the neurons with significant interaction(s), simple main effects were used as a *post hoc* test to analyze firing rates (*p* < 0.05) in each specific condition depending on the interaction.

In the present study, the path segment to the T-reward area overlapped in the left and right routes (overlapping path condition), whereas the path segments to L- and R-reward areas did not overlap (non-overlapping path condition). Previous fMRI studies reported that overlapping items were more distinctly represented than non-overlapping items in the human HF (Chanales et al., 2017; Kim et al., 2017). If pre-reward activity carries route information, we hypothesized that representation of the six path segments by ensemble pre-reward activity in the left and right routes across the three VN tasks would be more distinct in the overlapping, rather than the non-overlapping, path condition. To test this hypothesis, the mean correlation among population vectors consisting of ensemble pre-reward neuronal activity in the six path segments in the overlapping path condition was compared to that in the non-overlapping path condition (paired *t*-test, *p* < 0.05). Each pre-reward activity was normalized by mean firing rate in each VN task.

The 54 reward-related neurons were tested with the all three VN tasks, and these neurons responded different reward areas in the different VN tasks (see section “Results”). To analyze how these neurons remapped to different reward areas across the tasks, we analyzed the correlation of reward-related responses between two different tasks. There were 10 reward areas in the two routes in each task. In a given reward-related neuron, firing rates in the four 1-s bins around reward delivery was calculated in each reward area in each task. Then, correlation of reward-related responses across the 10 reward areas between given two tasks was computed in each reward-related neuron.

To analyze the spatial distribution of locations where activity of reward-related and place-related neurons increased, averaged firing rates maps were separately created in place- and reward-related neurons in the control VN task. First, firing rates in each pixel were normalized by scaling the minimum and maximum values to 0 and 1, respectively, (min-max feature scaling normalization) in each neuron (Munn and Bilkey, 2012; Royer et al., 2012). Then, averaged firing rate maps of place- and reward-related neurons were created separately.

### Bayesian Decoding

We used Bayesian decoding to check whether the population activity of route-dependent neurons in the central common hallway can predict the direction of the turn (i.e., route) (Zhang et al., 1998; Quiroga and Panzeri, 2009). The Bayesian decoding method computes the posterior probability of the turn direction D given spike counts S, p(D| S). The prediction by the decoder for given spike counts S is defined as the D maximizing p(D| S). The input data of Bayesian decoding were derived from the route coding neurons with significant route effects using a two-way ANOVA. We used route-dependent neurons that were tested in more than 10 trials for each route (*n* = 7) in the control VN task.

To test prediction accuracy of the decoder, we used “leave-one-out” validation, which allows to efficiently create the decoder and test with a small number of trials as small as 6 trials (Bower et al., 2005; Quiroga et al., 2007; Quiroga and Panzeri, 2009). To validate the decoder, data used for optimizing the decoder (training data) and evaluating the performance (test data) were separated to prevent an artificially high performance. In the “leave-one-out” validation, all data, except those from one trial, were used for training [i.e., optimizing p (D| S)]; the prediction was tested for the remaining trial. The process was repeated to calculate the prediction of all trials. Further, the percentage of correct predictions was calculated as the prediction accuracy. This process ensured that the largest possible number of trials could be utilized to train and test the decoder.

To test whether prediction accuracy was significantly larger than chance level, a “bootstrap” procedure was used to estimate the chance distribution (Bower et al., 2005). In this procedure, the direction memberships of each population neural activity were randomized, and the prediction accuracy was calculated. The chance distribution of the accuracy was obtained by repeating this process 10,000 times. In addition to route decoding, data matrix of mean firing rates in the three zones between T and C reward areas (zones 1, 2, and 3) were similarly submitted to zone decoding in each route.

We also examined whether responses to reward delivery in the common central hallway would include route information as well as temporal information of reward delivery. To decode this information from reward responses, HF neurons that had significant reward responses (*p* < 0.05, one-way ANOVA), at least at the T and/or C reward areas, in either routes were identified. Mean firing rates of these neurons, which showed not only significant reward responses at the T-reward area (*p* < 0.05, one-way ANOVA) but also significant activity changes during the pre-reward period (i.e., during the 2 s before reward delivery) from the mean firing rates (*p* < 0.05, one sample *t*-test), during the 2-s period before reward delivery at the T reward area in both routes were submitted to route decoding. In the same way, mean firing rates of the neurons, which showed not only significant reward responses at the C-reward area (*p* < 0.05, one-way ANOVA) but also significant activity changes during the post-reward period (i.e., during the 2 s after reward delivery) from the mean firing rates (*p* < 0.05, one sample *t*-test), during the 2-s period after reward delivery at C reward area in both routes were submitted to route decoding. Further, mean firing rates during the 2-s period before and after reward delivery at the T and C reward areas in both routes were separately submitted to temporal decoding of reward delivery in the same way.

### Neural Correlates to Saccades

Instantaneous speeds of eye movements were calculated from the X-Y positions of the eye. The rapid onset of eye movements, which were defined as an eye movement amplitude exceeding the experimenter-defined threshold (i.e., 0.3 mm/s), were identified. The minimum interval between two saccades was set at 100 ms; all eye movements within 100 ms from preceding saccades were ignored. The effect of saccades on HF neuronal activity within place fields were analyzed by creating peri-event histograms around saccade onsets. The significance of the saccade modulation was determined by comparing the neuronal activity between the 125-ms pre- and post-periods using a *t*-test (*p* < 0.05). Of 57 place-related neurons, one neuron displayed significant correlations to saccades inside place fields (Supplementary Figure 1).

A previous study reported vicarious trial and error (VTE)-like behaviors around a choice point in rodents (Johnson et al., 2007). To investigate possible VTE-like eye movements, we analyzed number of saccades around zones 3 and 2 before the choice point (T reward area) in each recording session for each task; number of saccades during 1 s just before reward delivery at T reward area (1 s from −1 to 0 s before the reward delivery at T reward area) was compared with that during a control period for 1 s from −2 to −1 s before reward delivery at T reward area (*p* < 0.05, two sample *t*-test). The results indicated that, only in four sessions out of a total of 66 sessions, there were significant differences in number of saccades between the 2 periods (*p* < 0.05). A previous study reported that VTE can be observed during the learning and early exposure to a task (Johnson et al., 2007), and this lack of VTE-like eye movements in the present study might be ascribed to the fact that the tasks were highly familiar to the monkeys since the monkeys were repeatedly trained until the correct response rate exceeded 99% in the present study. Although four neurons were found during these four sessions with significant differences in number of saccades, these four neurons showed no reward-related nor place-related responses before or in the choice point.

### Stereotaxic Localization of the Recording Sites

Before the start of each recording session, a three-dimensional magnetic resonance imaging (3-D MRI) scan of the monkey’s head was performed (Hori et al., 2005). The locations of HF neurons were based on the zero coordinates defined in the stereotaxic atlas of the Macaca fuscata brain (Kusama and Mabuchi, 1970).

## Results

### HF Place-Related Activity

**FIGURE 2:**
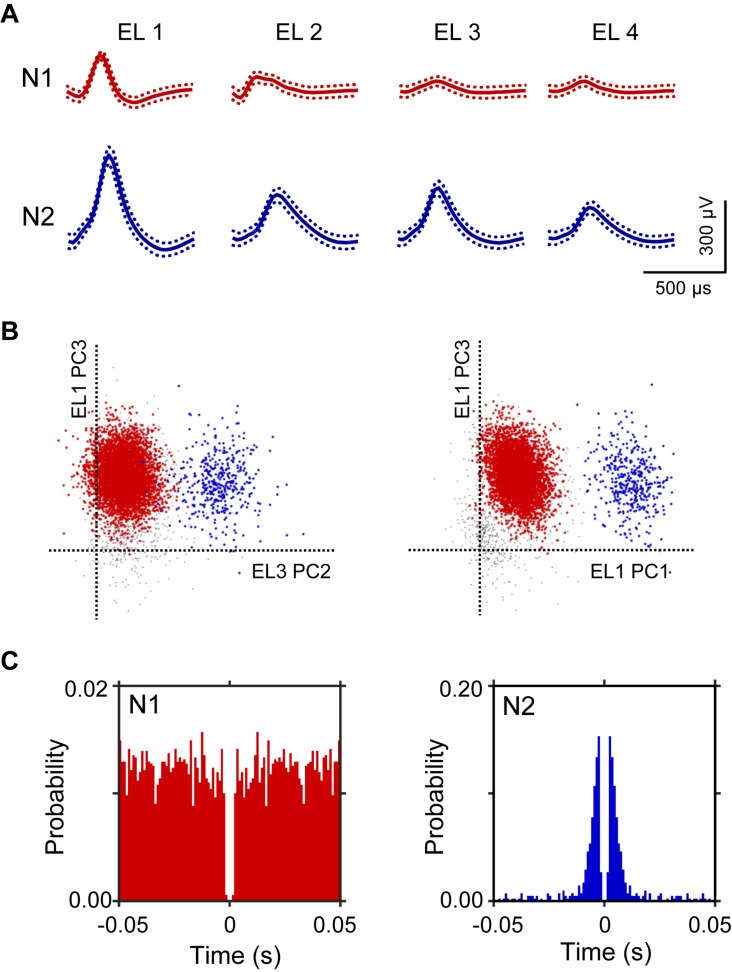
An example of an offline spike sorting. **(A)** Waveforms (mean ± standard deviation, indicated by solid and dotted lines, respectively) recorded from four electrodes (tetrode; EL 1–4). The N1 (red) and N2 (blue) waveforms correspond to two individual neurons, identified by offline spike sorting in B. **(B)** Results of offline spike sorting. Each axis represents a principle component (PC1-3). Each dot represents one neuronal spike. Semi-automatic spike sorting resulted in two clusters of neuronal signals (red and blue dots, corresponding to N1 and N2 in A, respectively). Gray dots represent unsorted spikes. **(C)** Autocorrelograms of the neurons. Bin width = 1 ms. Ordinates indicate probability, where bin counts were divided by the number of spikes in the spike train.

A total of 106 neurons were recorded from the left and right HF. Figure 2 shows an example of the raw signal of a HF neuron. Typical waveforms, which were simultaneously recorded from the same tetrode (EL 1–4), of two HF neurons (N1 and N2) are shown in Figure 2A. Figure 2B displays the results of spike sorting using offline cluster cutting of neuronal activities shown in Figure 2A. Each dot represents one spike. Two clusters of dots, which are indicated by different colors, were recognized. The autocorrelograms of these neurons indicated that their refractory periods were more than 1 ms, which suggests that these spikes were recorded from single neurons (Figure 2C).

**FIGURE 3:**
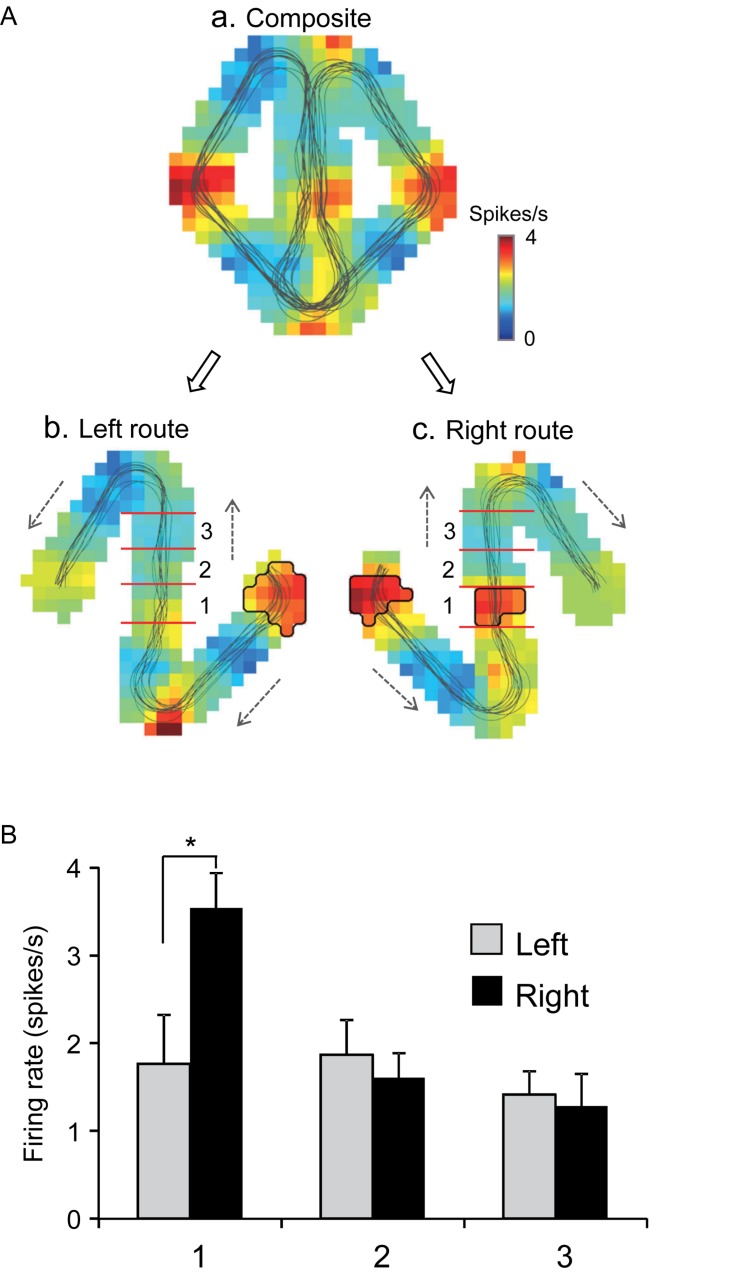
An example of a route-dependent place-related neuron in the VN task. **(A)** Firing rate maps. Place fields were identified near the R-reward area in the left route (b), and near the L- and C-reward areas in the right route (c). Trajectories of the monkey are indicated by thin lines on the firing rate maps. The place fields are marked using thick lines. **(B)** Comparison of mean firing rates in the three zones (1–3) in the left and right routes. ^∗^*p* < 0.05.

**FIGURE 4:**
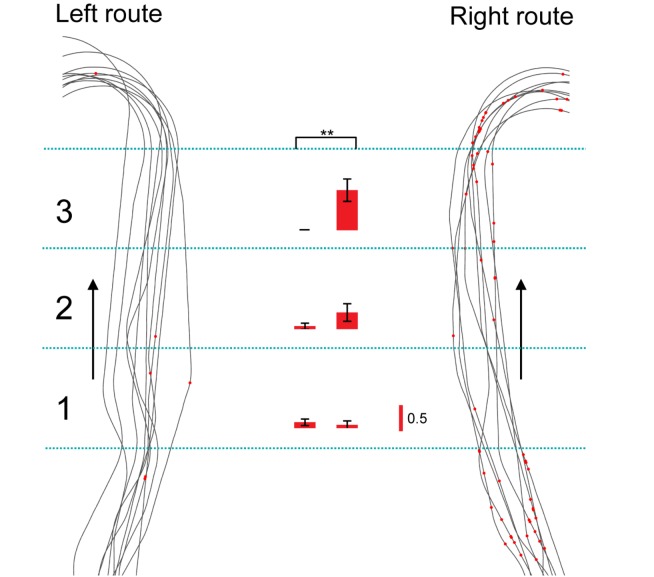
Another example of a route-dependent neuron in the VN task. Red dots on trails indicate neuronal spikes. Left and right histograms indicate mean firing rates in the three zones in the common central hallway (1–3) in the left and right routes, respectively. Calibration in the right bar indicates 0.5 spikes/s. ^∗∗^*p* < 0.01.

Of the 106 neurons recorded, 57 (53.8%) neurons displayed place field(s) in at least one of three VN tasks (place-related neurons) (Table 1). Figure 3 shows an example of a HF place-related neuron. The activity of the neuron increased around reward areas (Figure 3Aa). Place field analysis in the separate routes indicated place fields around the R-reward area in the left route (Figure 3Ab) and the C- and L-reward areas in the right route (Figure 3Ac). Moreover, this neuron demonstrated route-dependent activity. It displayed a place field in zone 1 in the common central hallway in the right route (Figure 3Ac), while no place field was recognized in the corresponding area in the left route (Figure 3Ab). Figure 3B shows mean firing rates in the three zones of the left and right routes. A two-way ANOVA indicated that there was a significant interaction between route and zone in the common straight path segment [*F*(2,55) = 6.8263, *p* = 0.0220]. *Post hoc* comparisons indicated that activity in the zone 1 of the right route was significantly larger than that in the left route (Bonferroni test, *p* < 0.05). Another example of a route-dependent activity is shown in Figure 4. A two-way ANOVA indicated that there was a significant effect of route [*F*(1,61) = 1.5033, *p* = 0.0029]. A total of 18 neurons displayed similar route-dependent activity in the common central hallway between the C and T reward areas (Table 1).

**TABLE 1:** Classification and number of hippocampal (HF) neuronal activity.

	**Total**	**Control**	**Rotated**	**No-flag**
Tested neurons	106	106	57	57
Place-related	57	43	26	25
Route-dependent	18	12	5	1
Reward-related	75	65	35	39
Route-dependent reward-related	56	47	24	24

*Control, control virtual navigation (VN) task; Rotated, rotated VN task; No-flag, no-flag VN task.*

**FIGURE 5:**
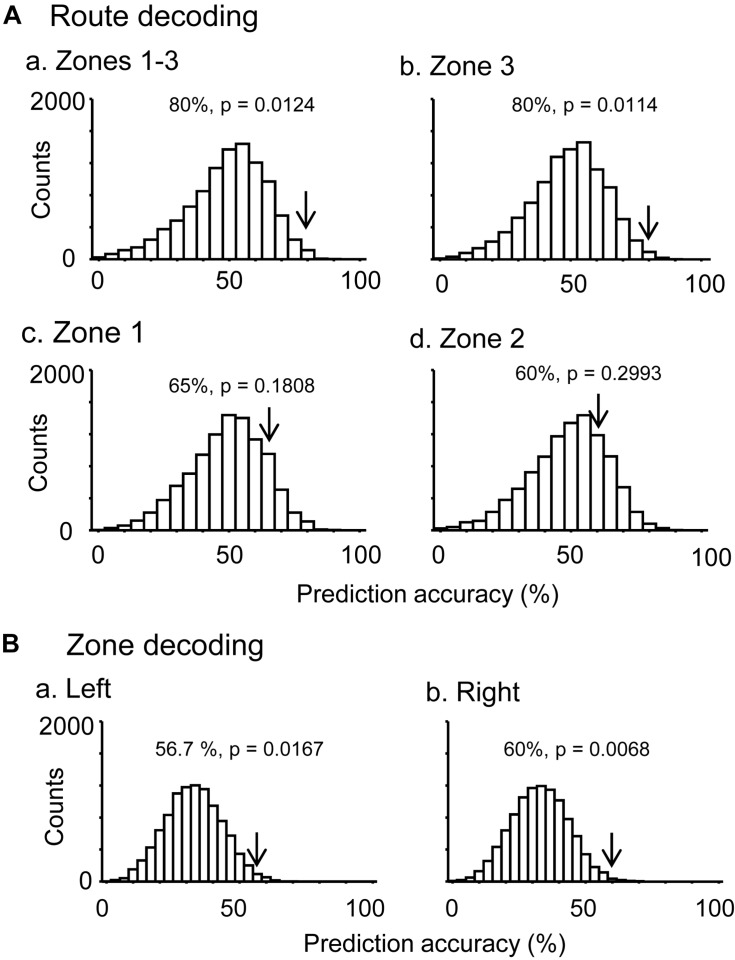
Bayesian decoding of overlapping path segments from ensemble place-related activity. **(A)** Route decoding from place-related activity in zones 1–3 (a), zone 3 (b), zone 1 (c), and zone 2 (d). **(B)** Zone decoding from place-related activity in the left (a) and right (b) routes. The arrow in each histogram indicates the actual prediction accuracy, while the histogram indicates the chance level distribution obtained using the “bootstrap” procedure (10,000 repetitions). The *p*-value for each prediction accuracy was calculated as the ratio of the count of the values ≥ the actual prediction accuracy in the chance.

In the control VN task, there were seven neurons that were tested in more than 10 trials in each route, which showed route-dependent activity in the common central hallway. When the mean firing rates in the three zones (zones 1–3) were used for route decoding, the ensemble activity of these seven neurons significantly predicted route (*p* < 0.05) (Figure 5Aa). The ensemble activity of these seven neurons also significantly predicted route when the data were confined to those in zone 3 (Figure 5Ab). However, neither the ensemble data in zone 1 (*p* > 0.05) nor those in zone 2 (*p* > 0.05) significantly predicted the route (Figures 5Ac,d). Moreover, ensemble activity of these seven neurons significantly predicted zone (*p* < 0.05) when mean firing rates in the three zones of the left route were submitted to zone decoding (Figure 5Ba). When the mean firing rates in the three zones of the right route were submitted to zone decoding, the ensemble activity of these seven neurons significantly predicted zone (*p* < 0.01) (Figure 5Bb). These results indicated that ensemble activity of the route-dependent neurons in the common central hallway conveyed route and zone information. We also submitted all the neurons which were tested in more than 10 trials, regardless of response types (*n* = 57), to route decoding analyses, but no ensemble data predicted the route (data not shown).

### Neural Correlates to Reward Delivery

**FIGURE 6:**
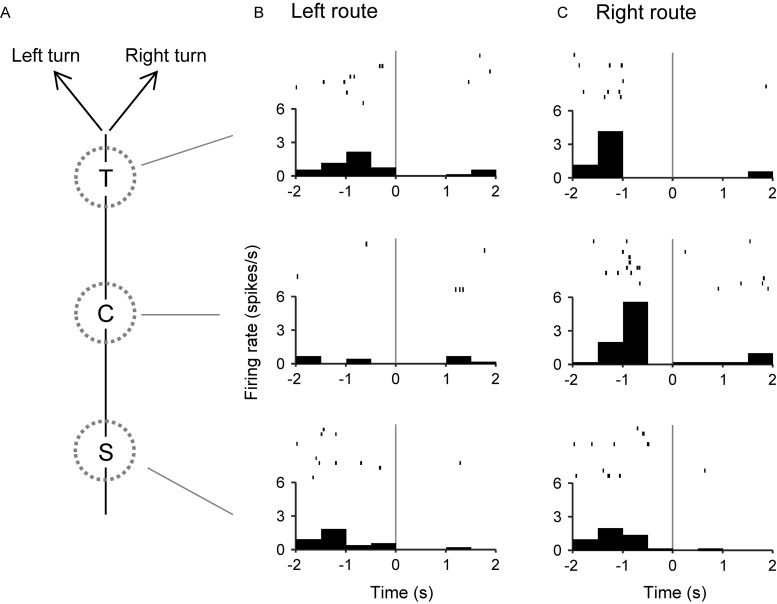
An example of a route-dependent reward-related neuron in the VN task. **(A)** Schematic illustration of the left and right routes in the common central hallway. **(B,C)** Three peri-event time histograms and raster plots around reward delivery at the T-, C-, and S-reward areas in the left **(B)** and right **(C)** routes. Gray lines indicate reward delivery.

The 57 place-related neurons displayed place field(s) in various area(s), some of which overlapped the reward areas in 36 place-related neurons. Regardless of place field(s), however, the activity of 75 neurons were modulated by reward delivery, in at least one of reward areas (reward-related neurons), in at least one of the three VN tasks (Table 1). These 75 neurons showed reward responses in some but not in all reward areas. In fact, there were no neuron that showed reward-related responses in every reward areas. Moreover, reward-related activity of 56 neurons in the common central hallway was modulated by route (route-dependent reward-related neurons) (Table 1). Figure 6 shows an example of a route-dependent reward-related HF neuron in the common central hallway. A three-way ANOVA indicated that there was a significant interaction among route, reward area, and period [*F*(6,218) = 2.4098, *p* = 0.0283].

**FIGURE 7:**
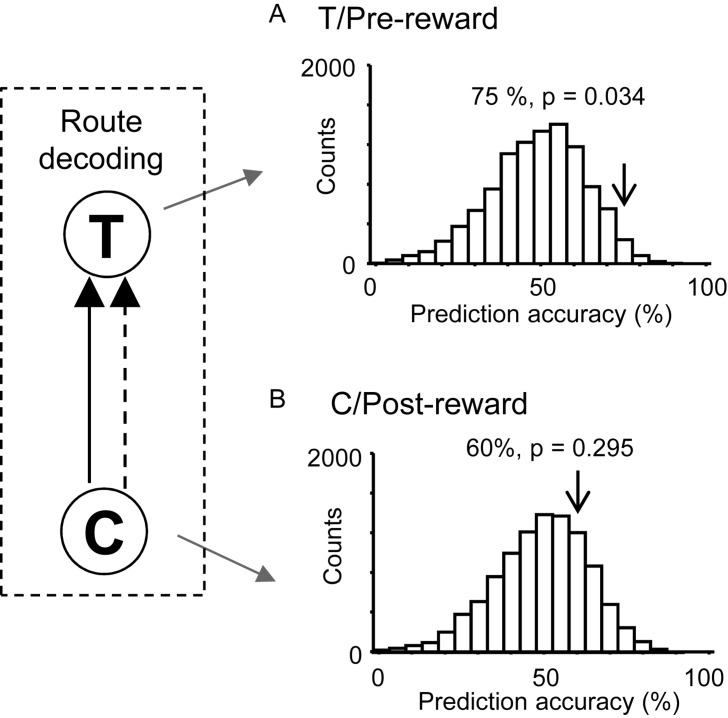
Bayesian decoding of overlapping path segments from reward-related activity in the control VN task. **(A)** Route decoding from pre-reward activity before reward delivery at the T-reward area. **(B)** Route decoding from post-reward activity after reward delivery at the C-reward area. The arrow in each histogram indicates the actual prediction accuracy, while the histogram indicates the chance level distribution obtained using the “bootstrap” procedure (10,000 repetitions). The *p*-value for each prediction accuracy was calculated as the ratio of the count of the values ≥ the actual prediction accuracy in the chance.

We hypothesized that responses to reward delivery would include route and temporal information. In the T-reward area in the control VN task, there were eight neurons that showed significant reward modulation (*p* < 0.05, one-way ANOVA) and significant activity changes during the pre-reward period (i.e., during the 2 s before reward delivery) from the mean firing rates (*p* < 0.05, one sample *t*-test). The ensemble activity of these eight neurons in the pre-reward period in the T-reward area was submitted to route decoding (Figure 7A). The results indicated that ensemble activity of these eight reward-related neurons with pre-reward responses significantly predicted route before reward delivery (*p* < 0.05). We also analyzed activity of reward-related neurons in the C-reward area in the same way, and there were five reward-related neurons with post-reward responses in the C-reward area in the control VN task. However, ensemble data in the post-reward period in the C-reward area did not significantly predict route (*p* > 0.05) (Figure 7B). In the other VN tasks, the ensemble data in the T-reward area tended to predict route in the rotated VN task (*p* < 0.1) and significantly predicted route in the no-flag VN task (*p* < 0.01).

**FIGURE 8:**
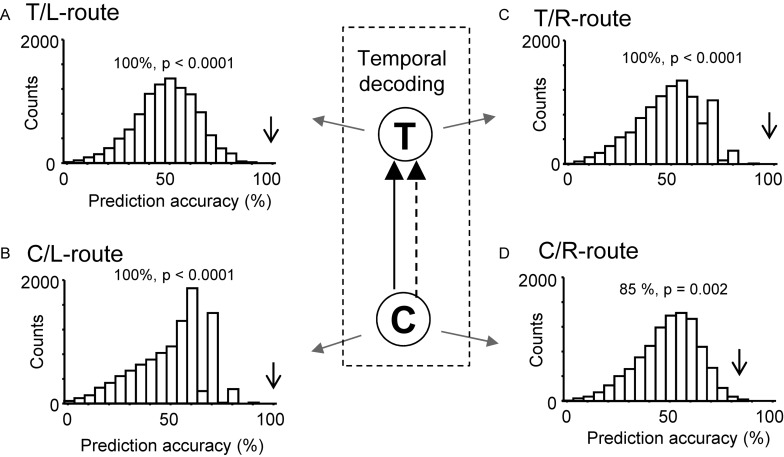
Bayesian decoding of reward delivery from reward-related activity in the control VN task. **(A,B)** Temporal decoding from reward-related activity around the T- (A) and C- (B) reward area in the left route. **(C,D)** Temporal decoding from reward-related activity around the T- (C) and C- (D) reward area in the right route. The arrow in each histogram indicates the actual prediction accuracy, while the histogram indicates the chance level distribution obtained using the “bootstrap” procedure (10,000 repetitions). The *p*-value for each prediction accuracy was calculated as the ratio of the count of the values ≥ the actual prediction accuracy in the chance.

Moreover, ensemble activity of the reward-related neurons predicted reward delivery at the T- and C-reward areas (Figure 8). The ensemble activity of 25 neurons, which showed reward-related activity at the T-reward-area in the left route (*p* < 0.05, one-way ANOVA), significantly predicted reward delivery (*p* < 0.0001) when mean firing rates during the 2-s periods before and after reward delivery in the T-reward area of the left route were submitted to temporal decoding in the control VN task (Figure 8A). In the same way, the ensemble activity of 14 neurons, which showed reward-related activity at the C-reward-area in the left route (*p* < 0.05, one-way ANOVA), significantly predicted reward delivery at the C-reward area in the left route (*p* < 0.0001) (Figure 8B) and T- and C-reward areas in the right route (*p* < 0.0001, 0.01, respectively) (Figures 8C,D). In the rotated and no-flag VN tasks, the decoding analyses of temporal relations showed comparative results (data not shown).

**FIGURE 9:**
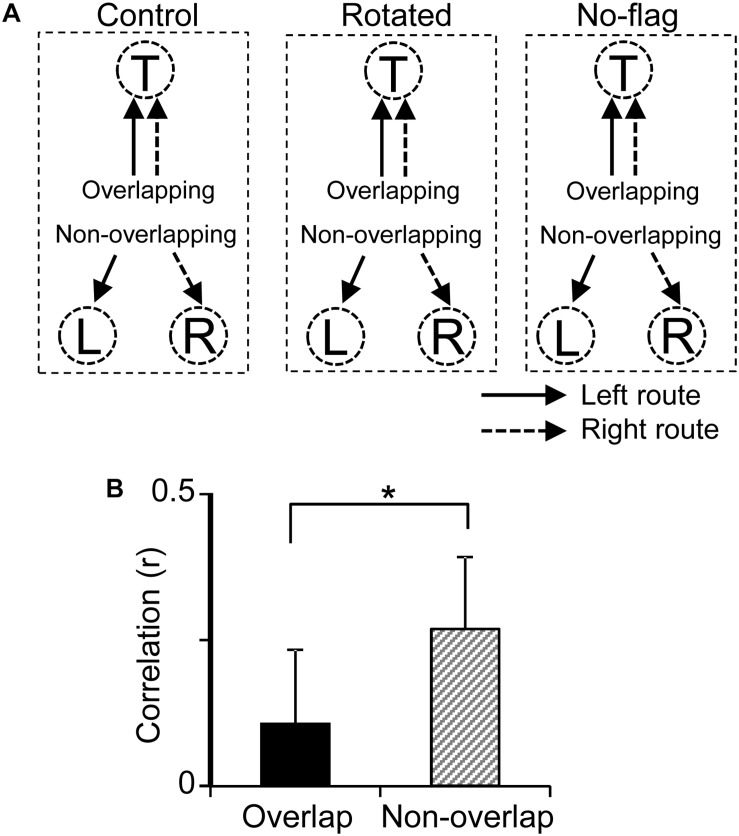
Representation of the overlapping and non-overlapping path segments by pre-reward activity of the reward-related neurons across the VN tasks. **(A)** Schematic illustration of overlapping and non-overlapping path conditions in the three VN tasks. **(B)** Comparison of mean correlation coefficients across the six path segments between the overlapping and non-overlapping path conditions. ^∗^*p* < 0.05.

Previous fMRI studies have reported that overlapping items were more distinctly represented than non-overlapping items in the human HF (Chanales et al., 2017; Kim et al., 2017). In the present study, the path segment to the T-reward area overlapped in the left and right routes (overlapping path condition), whereas the path segments to L- and R-reward areas did not overlap (non-overlapping path condition) (Figure 9A). The above results suggest that pre-reward activity carries route information, which further suggest that the representation of the six path segments by ensemble pre-reward activity in the left and right routes across the three VN tasks would be more distinct in the overlapping path condition than the non-overlapping path condition. A total of 16 neurons showed reward correlates in the T-reward area in one of the three VN tasks at least, whereas 20 neurons showed reward correlates in the L- and/or R-reward areas in one of the three VN tasks at least. The mean correlation among population vectors consisting of these 16 and 20 neuronal activities are shown in Figure 9B. The mean correlation was significantly smaller in the overlapping path condition than in the non-overlapping path condition (paired *t*-test, *p* < 0.05).

A recent rodent study has reported that some HF neurons specialized for encoding reward location are active not only in one environment but also across multiple environments (Gauthier and Tank, 2018). In the present study, 54 out of 57 HF neurons tested with the all three VN tasks showed reward-related responses in at least one of the three VN tasks. Of these 54 reward-related neurons, 15 (27.8%) neurons showed reward-related responses only one of the three VN tasks while 39 (72.2%) neurons showed reward-related responses in the multiple VN tasks. Across the different VN tasks, 23 of these 39 reward-related neurons were active in some of the same (but not all) reward areas while the remaining 16 neurons were active in different reward areas. Thus, no reward-related neurons showed identical reward-related responses across the 3 tasks. Consistently, correlation of reward-related responses across 10 reward areas in the two routes between the given two mazes was low: correlation between the control and no-flag VN tasks, 0.061 ± 0.023; correlation between the control and rotated VN tasks, 0.082 ± 0.022; correlation between the no-flag and rotated VN tasks, 0.108 ± 0.018. These findings indicate that the reward-related neurons remapped to different reward areas across the different VN tasks.

### Relationships Between Place-Related and Reward-Related Responses

**FIGURE 10:**
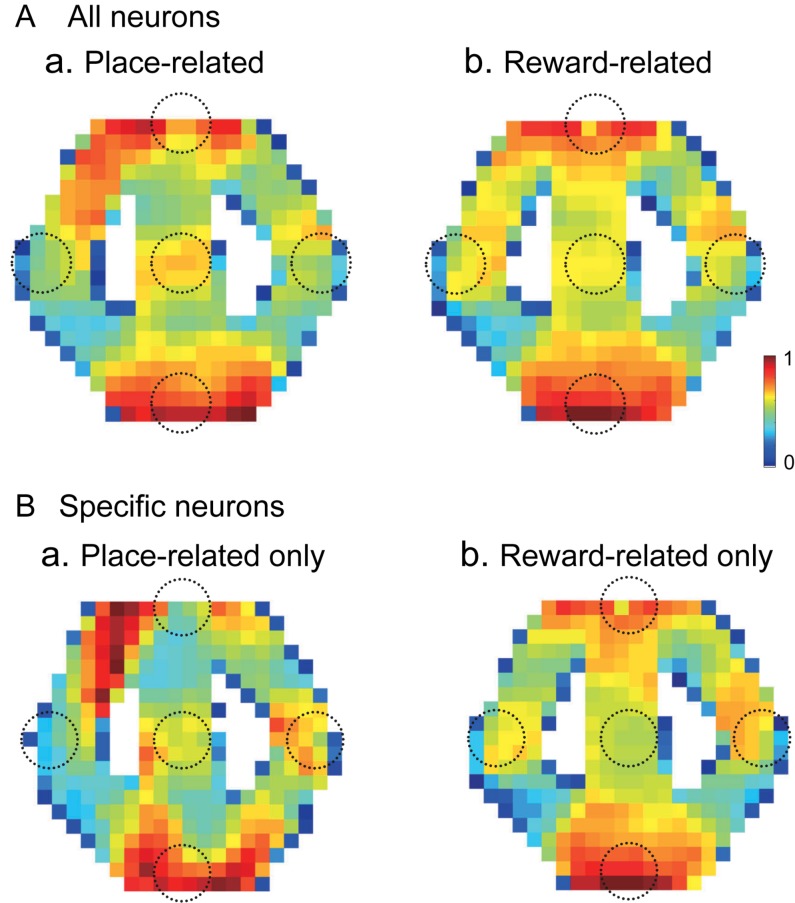
Averaged firing rate maps of the place- and reward-related neurons in the control VN task. **(A)** Averaged firing rate maps of the place-related (*n* = 43) (a) and reward-related (*n* = 65) (b) neurons when the all neurons in each category were analyzed. **(B)** Averaged firing rate maps of the place-related neurons without reward-related responses (*n* = 12) (a) and reward-related neurons without place-related responses (*n* = 34) (b).

Out of 57 place-related neurons, 49 showed reward-related responses in certain reward areas. Out of 75 reward-related neurons, 49 showed place-related responses in certain areas. Figure 10 shows averaged firing rate maps of place-related and reward-related neurons in the control VN task. When the data of the all place-related (*n* = 43) and reward-related (*n* = 65) neurons were analyzed (Figure 10A), activity of the neurons was increased around the T and S reward areas in both place-related and reward-related neurons. The firing rate maps of these two types of HF neurons were highly correlated (*r* = 0.872). There were 12 place-related neurons without reward-related responses and 34 reward-related neurons without place-related responses in the control VN task. When the data analyses were confined to these specific types of the HF neurons (Figure 10B), the averaged firing rate maps showed similar trends in the place-related neurons without reward-related neurons (Figure 10Ba) and reward-related neurons without place-related neurons (Figure 10Bb). The firing rate maps of these two types of HF neurons still showed high spatial correlation (*r* = 0.659).

### Recording Sites

**FIGURE 11:**
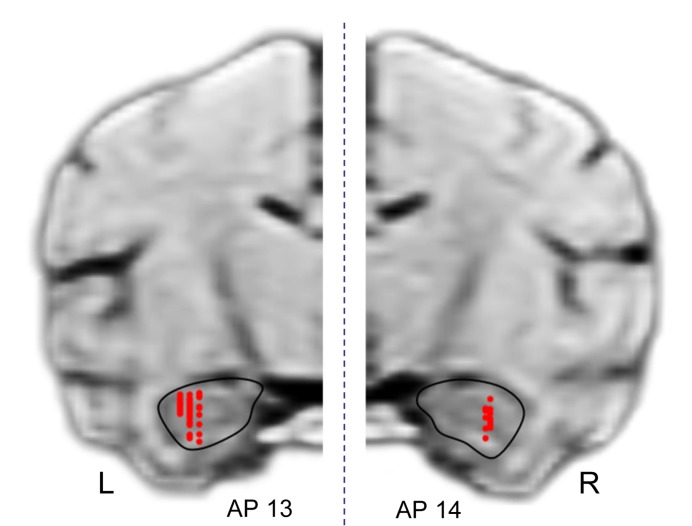
Recording sites in the hippocampal formation (HF). A total of 125 penetrations were performed in the HF. Red dots indicate recording sites of HF neurons. R, right hemisphere; L, left hemisphere.

Figure 11 shows the recording sites in the HF indicated by red dots. A total of 125 penetrations were performed in both sides of HF, and 49 neurons were recorded in the left HF (monkey A) while 57 neurons, in the right HF (monkey B) (indicated by red dots). In both sides of the HF, comparative numbers of place-related neurons (left HF, *n* = 16; right HF, *n* = 25) and reward-related neurons (left HF, *n* = 26; right HF, *n* = 31) were found in the control VN task. Statistical analyses indicated that there were no significant differences in ratios of place-related and reward-related neurons between the left and right HF (*p* > 0.05 for both place-related and reward-related neurons, Fisher’s exact test). Based on the stereotaxic atlas of the monkey brain (Kusama and Mabuchi, 1970), these recording sites correspond to CA1 and CA3 subfields, and dentate gyrus in both right and left HF. These results indicated that the data were comparable between the left and right sides of the HF.

## Discussion

### Place-Related Activity in the HF

In the present study, consistent with previous studies in human and non-human primates (Ono et al., 1993; Nishijo et al., 1997; Matsumura et al., 1999; Ekstrom et al., 2003; Hori et al., 2005; Miller et al., 2013; Furuya et al., 2014), place-related activity was observed in the monkey HF. Further, a total of 18 HF neurons displayed activity in the common central hallway, which was modulated by route. The decoding results suggest that the ensemble activity of these neurons carries information of route and monkey’s location in the three zones. During navigation in the common central hallway before turning (path segment between T- and C-reward areas), the monkey always faced the same visual cues, suggesting that this route-dependent activity was not ascribed to view differences of the monkey but to the routes. Consistent with previous rodent studies in which animals navigated the same path segment in specific direction(s) in different routes (Frank et al., 2000; Wood et al., 2000; Ferbinteanu and Shapiro, 2003; Dayawansa et al., 2006), the current study provided evidence that the activity of primate HF neurons is also route-dependent. A previous study on monkeys reported that place fields of place-related neurons were dependent on the size of virtual spaces (Furuya et al., 2014), consistent with a rodent study (O’Keefe and Burgess, 1996). Furthermore, a recent neurophysiological study in monkeys reported a similar type of HF neurons that responded differently when the subject rotated to the same direction in the same position but in different action contexts (Wirth et al., 2017), indicating the context-dependency in this HF neuronal responses. These findings extend previous findings in rodents by demonstrating the disambiguation of overlapping path segments in non-human primates.

In a previous study in rats, 10 (43.4%) of 23 route-dependent place-differential neurons still showed route-dependent place responses while animals were passively dislocated without locomotion, while the rest of these neurons were sensitive to locomotion (Dayawansa et al., 2006). Since the activity of the 18 neurons was dependent on the routes in the VN without locomotion in this study, it is suggested that such neurons in primates and rodents might code visual information (e.g., optic flow and landmarks) to form route-dependent activity.

Human fMRI studies reported that the HF is important and active when subjects navigate overlapping path segments in a virtual space (Brown et al., 2010; Chanales et al., 2017). The present decoding results indicated that route coding was more evident in zone 3 near the bifurcation point than in zones 1 and 2. This suggests that ensemble HF neuronal activity in zone 3 carries important information to reach reward goals (L- and R-reward areas). Rodent studies reported that ensemble activity near choice points carried prospective information to reach goals (Johnson and Redish, 2007; Catanese et al., 2014), but such activity was less observable in the same location when discrimination of route was not required (Griffin et al., 2012). Consistent with the results of the present study and previous rodent studies, a computational study also suggested that episodic memory regarding routes is used to navigate by cueing retrieval at the choice point (Zilli and Hasselmo, 2008). These findings suggest that route-dependent activity in zone 3 might be involved in future behavioral decision, and that disambiguation of the same path segment in the same environment is a hallmark of the HF in episodic memory (Griffin and Hallock, 2013; Eichenbaum, 2017).

### Reward-Related Activities

In the present study, 75 neurons showed neural correlates to reward delivery (reward-related neurons in Table 1), which was consistent with previous studies suggesting that reward is one of the important determinants of the neuronal activities in the monkey HF (Ono et al., 1993; Rolls and Xiang, 2005). Furthermore, the averaged firing rate maps indicated that place-related neurons without reward-related responses showed activity increases around reward areas similar to those in the reward-related neurons. Such accumulation of place fields around the goal (reward) areas has been reported in rodents as well (Hollup et al., 2001; Kobayashi et al., 2003; Hok et al., 2007). A similar phenomenon was observed in the current study; about 30% (36/106) of the HF neurons displayed their place fields overlapping with the reward (goal) areas, which was comparative to previous studies (Hollup et al., 2001; Kobayashi et al., 2003; Hok et al., 2007). The HF is implicated in reward-related functions, including place-reward association, conditioned place preference, and reinforcement learning (Hampton et al., 2004; Rolls and Xiang, 2005; Ito et al., 2008; Ho et al., 2011; Davidow et al., 2016), which might be a neural basis of drug addiction (Trouche et al., 2016; Xia et al., 2017). Neurons in the HF with goal (reward)-directed activity project to the nucleus accumbens, which plays a role in motivation and reward processing (Ciocchi et al., 2015), and reward-induced synchronization of neuronal activity between the HF and nucleus accumbens (Tabuchi et al., 2000). This HF-accumbal connection has also been implicated in reward memory in humans (Davidow et al., 2016).

The current study also indicated that HF neural correlates to reward delivery was route-dependent in the common central hallway. During the VN tasks, all reward (goal) areas provided the animals with a reward if the animals visited them in the correct order. This suggests that the association between reward (or reward expectation) and specific places in space could be the cause of the observed goal responses (Hölscher et al., 2003; Hok et al., 2005). Therefore, we postulated that HF neuronal activity around reward delivery would carry spatial information required for the navigation in the overlapping path segment in the same environment. Consistent with our hypothesis, route information was decoded from ensemble neuronal activity before reward delivery at the T-reward area. Further, the decoding analysis suggests that ensemble activity in each route carries temporal information of reward delivery. Reward delivery (i.e., reward outcome) is one of the important factors in episodic memory (Mason et al., 2017); it modulates HF neuronal activity (Lee et al., 2012; Tryon et al., 2017; Boccara et al., 2019). These findings suggest that the HF plays an important role in disambiguation of overlapping action (navigation)-reward outcome association. Furthermore, about 30% of reward-related neurons showed reward-related responses in only one of the three VN tasks while about 70% of reward-related neurons showed reward-related responses in the multiple tasks. The former type of the reward-related neurons might code task (context)-specific information of reward areas, while the latter type might simply code reward events (Gauthier and Tank, 2018). In spite of the remap to new reward areas across the different VN tasks in reward-related neurons, activity of these neurons predicted routes and reward delivery. Taken together, these results suggest that HF neuronal activity carries multiple information, including route, outcome (reward delivery), and context information. The present results extend the role of the HF to disambiguation of reward outcome, consistent with the role the HF in episodic memory.

Consistent with the present decoding findings suggesting that route-dependent reward-related neurons contribute to navigation, activity of reward-related neurons was reported to be correlated to spatial memory performance in rodents (Dupret et al., 2010). This may suggest that the relatively small number of route-dependent neurons compared with reward-related neurons in the present study might be ascribed to the task design. This may also explain the insignificant results in the decoding analysis using whole sample neurons. In many previous studies in rodents, the reward was provided at the end of a trajectory, while in this study, the entire path could be segmented by each reward areas along the route. Thus, the route-dependent reward-related neurons could provide sufficient information for navigation to a next reward area in the present study, and consequently information from route-dependent (reward non-related) neurons might not be fully required for navigation in such a path segment. The present results are consistent with a previous study (Bower et al., 2005), in which rats were trained to trace reward areas in sequence and route-dependent activity was not evident compared with the other rodent studies (see section “Introduction”). However, route-dependent (reward non-related) place cells might be required to correctly navigate an overlapping path segment that is not segmented by reward areas (e.g., Wood et al., 2000). In such cases, combination of route-dependent place cells and a set of goal cells might code a specific route to that final destination (Burgess and O’Keefe, 1996). Monkey HF neurons were sensitive to multiple variables (Wirth et al., 2017), with neurons encoding egocentric and allocentric references in relation to the surrounding environment. The present findings add reward as another encoded factor when it has relevance for spatial navigation (as a waypoint). Consistently, the place-related neurons without reward-related responses also showed similar activity increases around the reward areas as did the reward-related neurons.

In addition, representation of the six path segments by ensemble pre-reward activity were more distinct in the overlapping path condition (pre-reward activity in the common path segments from the C to T reward areas) than in the non-overlapping path condition (pre-reward activity in path segments from the T to L or R reward areas) (Figure 9). These results also corroborate with a previous human fMRI study, in which overlapping path segments or objects were more distinctly represented than non-overlapping path segments or objects (Hulbert and Norman, 2015; Chanales et al., 2017). In a computational model, known as “neural differentiation,” neuronal ensembles representing overlapping events (or items) become differentiated (separate neural ensembles) by competition due to repeated retrieval (Hulbert and Norman, 2015). The present results provide novel neurophysiological evidence supporting neural differentiation.

## Conclusion

The HF is implicated in disambiguation of overlapping spaces in rodents (Frank et al., 2000; Wood et al., 2000; Ferbinteanu and Shapiro, 2003; Dayawansa et al., 2006; Ainge et al., 2007; Grieves et al., 2016). Here we demonstrated this role in monkeys, by recording HF neuronal activity, while animals navigated Figure 8 pathways, including an overlapping common central hallway in a virtual space to acquire rewards. Route-dependent neural activity was observed in 18 neurons, with decoding data suggesting that the ensemble activity of these neurons carries information about route and the monkey’s location in the three zones of the common central hallway. Moreover, this role in disambiguation was observed to extend to reward acquisition, with 56 neurons showing route-dependent reward-related activity. Decoding data suggest that the ensemble activity of these neurons also carries information on route and reward delivery (outcome). Understanding the relations between memory, navigation, and reward may bring new insights into the role of the HF in addiction (Trouche et al., 2016; Xia et al., 2017) and motivation (Kennedy and Shapiro, 2009; Lebreton et al., 2013). Moreover, overlapping reward areas were more distinctly represented than the non-overlapping ones. These findings suggest an impact of reward on HF coding of overlapping items. Consistent with the present idea, overlapping stimuli associated with high rewards were less susceptible to interference, being associated with increased HF activity (Kuhl et al., 2010). Taken together, the present results may provide neural evidence that disambiguation of overlapping items is organized based on neural differentiation (Hulbert and Norman, 2015) in the non-human primate HF.

## Data Availability

The datasets generated for this study are available on request to the corresponding author.

## Ethics Statement

The animal study was reviewed and approved by the ethical committee for animal experiments in the University of Toyama.

## Author Contributions

HisN conceived the study and designed the experiments. RB performed the experiments. RB and JM analyzed the data and wrote the manuscript. HisN, HirN, JM, YT, EH, and TO revised the manuscript. All authors discussed the results and commented on the manuscript, and read and approved the final manuscript.

## Conflict of Interest Statement

The authors declare that the research was conducted in the absence of any commercial or financial relationships that could be construed as a potential conflict of interest.

## Footnotes

1 http://neurosuite.sourceforge.net/

2 http://klustakwik.sourceforge.net/

## References

[B1] Ainge J. A. Meer M. A. A. van der Langston R. F. Wood E. R. (2007). Exploring the role of context-dependent hippocampal activity in spatial alternation behavior. *Hippocampus* 17 988–1002. 10.1002/hipo.20301 17554771

[B2] Ainge J. A. Tamosiunaite M. Wörgötter F. Dudchenko P. A. (2012). Hippocampal place cells encode intended destination, and not a discriminative stimulus, in a conditional T-maze task. *Hippocampus* 22 534–543. 10.1002/hipo.20919 21365712

[B3] Boccara C. N. Nardin M. Stella F. O’Neill J. Csicsvari J. (2019). The entorhinal cognitive map is attracted to goals. *Science* 363 1443–1447. 10.1126/science.aav4837 30923221

[B4] Bower M. R. Euston D. R. McNaughton B. L. (2005). Sequential-context-dependent hippocampal activity is not necessary to learn sequences with repeated elements. *J. Neurosci.* 25 1313–1323. 10.1523/JNEUROSCI.2901-04.2005 15703385 PMC6725980

[B5] Bretas V. R. (2018). *Neural Representation of Overlapping Trajectories and Reward Acquisitions in the Monkey Hippocampus.* Available at: https://toyama.repo.nii.ac.jp/?action=pages_view_main&active_action=repository_view_main_item_detail&item_id=16103&item_no=1&page_id=32&block_id=36 (accessed April 1, 2018). 10.3389/fnsys.2019.00048 PMC6751269 31572133

[B6] Brown T. I. Ross R. S. Keller J. B. Hasselmo M. E. Stern C. E. (2010). Which way was I going? Contextual retrieval supports the disambiguation of well learned overlapping navigational routes. *J. Neurosci.* 30 7414–7422. 10.1523/JNEUROSCI.6021-09.2010 20505108 PMC2905880

[B7] Burgess N. Maguire E. A. O’Keefe J. (2002). The human hippocampus and spatial and episodic memory. *Neuron* 35 625–641. 10.1016/S0896-6273(02)00830-9 12194864

[B8] Burgess N. O’Keefe J. (1996). Neuronal computations underlying the firing of place cells and their role in navigation. *Hippocampus* 6 749–762. 10.1002/(sici)1098-1063(1996)6:6<749::aid-hipo16>3.0.co;2-0 9034860

[B9] Catanese J. Viggiano A. Cerasti E. Zugaro M. B. Wiener S. I. (2014). Retrospectively and prospectively modulated hippocampal place responses are differentially distributed along a common path in a continuous T-maze. *J. Neurosci.* 34 13163–13169. 10.1523/JNEUROSCI.0819-14.2014 25253861 PMC4172807

[B10] Chanales A. J. H. Oza A. Favila S. E. Kuhl B. A. (2017). Overlap among spatial memories triggers repulsion of hippocampal representations. *Curr. Biol.* 27 2307.e5–2317.e5. 10.1016/j.cub.2017.06.057 28736170 PMC5576038

[B11] Ciocchi S. Passecker J. Malagon-Vina H. Mikus N. Klausberger T. (2015). Selective information routing by ventral hippocampal CA1 projection neurons. *Science* 348 560–563. 10.1126/science.aaa3245 25931556

[B12] Davidow J. Y. Foerde K. Galván A. Shohamy D. (2016). An upside to reward sensitivity: the hippocampus supports enhanced reinforcement learning in adolescence. *Neuron* 92 93–99. 10.1016/j.neuron.2016.08.031 27710793

[B13] Dayawansa S. Kobayashi T. Hori E. Umeno K. Tazumi T. Ono T. (2006). Conjunctive effects of reward and behavioral episodes on hippocampal place-differential neurons of rats on a mobile treadmill. *Hippocampus* 16 586–595. 10.1002/hipo.20186 16685707

[B14] Dupret D. O’neill J. Pleydell-Bouverie B. Csicsvari J. (2010). The reorganization and reactivation of hippocampal maps predict spatial memory performance. *Nat. Neurosci.* 13 995–1002. 10.1038/nn.2599 20639874 PMC2923061

[B15] Eichenbaum H. (2017). The role of the hippocampus in navigation is memory. *J. Neurophysiol.* 117 1785–1796. 10.1152/jn.00005.2017 28148640 PMC5384971

[B16] Eichenbaum H. Stewart C. Morris R. G. (1990). Hippocampal representation in place learning. *J. Neurosci.* 10 3531–3542. 10.1523/JNEUROSCI.10-11-03531.1990 2230943 PMC6570096

[B17] Ekstrom A. D. Kahana M. J. Caplan J. B. Fields T. A. Isham E. A. Newman E. L. (2003). Cellular networks underlying human spatial navigation. *Nature* 425 184–188. 10.1038/nature01964 12968182

[B18] Ferbinteanu J. Shapiro M. L. (2003). Prospective and retrospective memory coding in the hippocampus. *Neuron* 40 1227–1239. 10.1016/S0896-6273(03)00752-9 14687555

[B19] Frank L. M. Brown E. N. Wilson M. (2000). Trajectory encoding in the hippocampus and entorhinal cortex. *Neuron* 27 169–178. 10.1016/S0896-6273(00)00018-0 10939340

[B20] Furuya Y. Matsumoto J. Hori E. Boas C. V. Tran A. H. Shimada Y. (2014). Place-related neuronal activity in the monkey parahippocampal gyrus and hippocampal formation during virtual navigation. *Hippocampus* 24 113–130. 10.1002/hipo.22209 24123569

[B21] Gauthier J. L. Tank D. W. (2018). A dedicated population for reward coding in the hippocampus. *Neuron* 99 179–193. 10.1016/j.neuron.2018.06.008 30008297 PMC7023678

[B22] Grieves R. M. Wood E. R. Dudchenko P. A. (2016). Place cells on a maze encode routes rather than destinations. *eLife* 5:e15986. 10.7554/eLife.15986 27282386 PMC4942257

[B23] Griffin A. L. Hallock H. L. (2013). Hippocampal signatures of episodic memory: evidence from single-unit recording studies. *Front. Behav. Neurosci.* 7:54. 10.3389/fnbeh.2013.00054 23734111 PMC3661991

[B24] Griffin A. L. Owens C. B. Peters G. J. Adelman P. C. Cline K. M. (2012). Spatial representations in dorsal hippocampal neurons during a tactile-visual conditional discrimination task. *Hippocampus* 22 299–308. 10.1002/hipo.20898 21080411

[B25] Hampton R. R. Hampstead B. M. Murray E. A. (2004). Selective hippocampal damage in rhesus monkeys impairs spatial memory in an open-field test. *Hippocampus* 14 808–818. 10.1002/hipo.10217 15382251

[B26] Harris K. D. Henze D. A. Csicsvari J. Hirase H. Buzsáki G. (2000). Accuracy of tetrode spike separation as determined by simultaneous intracellular and extracellular measurements. *J. Neurophysiol.* 84 401–414. 10.1152/jn.2000.84.1.401 10899214

[B27] Harvey C. D. Collman F. Dombeck D. A. Tank D. W. (2009). Intracellular dynamics of hippocampal place cells during virtual navigation. *Nature* 461 941–946. 10.1038/nature08499 19829374 PMC2771429

[B28] Hazama Y. Tamura R. (2019). Effects of self-locomotion on the activity of place cells in the hippocampus of a freely behaving monkey. *Neurosci. Lett.* 701 32–37. 10.1016/j.neulet.2019.02.009 30738872

[B29] Hazan L. Zugaro M. Buzsáki G. (2006). Klusters, NeuroScope, NDManager: a free software suite for neurophysiological data processing and visualization. *J. Neurosci. Methods* 155 207–216. 10.1016/j.jneumeth.2006.01.017 16580733

[B30] Ho A. S. Hori E. Nguyen P. H. T. Urakawa S. Kondoh T. Torii K. (2011). Hippocampal neuronal responses during signaled licking of gustatory stimuli in different contexts. *Hippocampus* 21 502–519. 10.1002/hipo.20766 20087892

[B31] Ho S. A. Hori E. Kobayashi T. Umeno K. Tran A. H. Ono T. (2008). Hippocampal place cell activity during chasing of a moving object associated with reward in rats. *Neuroscience* 157 254–270. 10.1016/j.neuroscience.2008.09.004 18824217

[B32] Hok V. Lenck-Santini P.-P. Roux S. Save E. Muller R. U. Poucet B. (2007). Goal-related activity in hippocampal place cells. *J. Neurosci.* 27 472–482. 10.1523/JNEUROSCI.2864-06.2007 17234580 PMC6672791

[B33] Hok V. Save E. Lenck-Santini P. P. Poucet B. (2005). Coding for spatial goals in the prelimbic/infralimbic area of the rat frontal cortex. *Proc. Natl. Acad. Sci. U.S.A.* 102 4602–4607. 10.1073/pnas.0407332102 15761059 PMC555486

[B34] Hollup S. A. Molden S. Donnett J. G. Moser M.-B. Moser E. I. (2001). Accumulation of hippocampal place fields at the goal location in an annular watermaze task. *J. Neurosci.* 21 1635–1644. 10.1523/JNEUROSCI.21-05-01635.2001 11222654 PMC6762966

[B35] Hölscher C. Jacob W. Mallot H. A. (2003). Reward modulates neuronal activity in the hippocampus of the rat. *Behav. Brain Res.* 142 181–191. 10.1016/S0166-4328(02)00422-9 12798280

[B36] Hori E. Nishio Y. Kazui K. Umeno K. Tabuchi E. Sasaki K. (2005). Place-related neural responses in the monkey hippocampal formation in a virtual space. *Hippocampus* 15 991–996. 10.1002/hipo.20108 16108028

[B37] Hulbert J. C. Norman K. A. (2015). Neural differentiation tracks improved recall of competing memories following interleaved study and retrieval practice. *Cereb. Cortex* 25 3994–4008. 10.1093/cercor/bhu284 25477369 PMC4585527

[B38] Ito R. Robbins T. W. Pennartz C. M. Everitt B. J. (2008). Functional interaction between the hippocampus and nucleus accumbens shell is necessary for the acquisition of appetitive spatial context conditioning. *J. Neurosci.* 28 6950–6959. 10.1523/JNEUROSCI.1615-08.2008 18596169 PMC3844800

[B39] Johnson A. Redish A. D. (2007). Neural ensembles in ca3 transiently encode paths forward of the animal at a decision point. *J. Neurosci.* 27 12176–12189. 10.1523/JNEUROSCI.3761-07.2007 17989284 PMC6673267

[B40] Johnson A. van der Meer M. A. Redish A. D. (2007). Integrating hippocampus and striatum in decision-making. *Curr. Opin. Neurobiol.* 17 692–697. 10.1016/j.conb.2008.01.003 18313289 PMC3774291

[B41] Kennedy P. J. Shapiro M. L. (2009). Motivational states activate distinct hippocampal representations to guide goal-directed behaviors. *PNAS* 106 10805–10810. 10.1073/pnas.0903259106 19528659 PMC2705558

[B42] Kim G. Norman K. A. Turk-Browne N. B. (2017). Neural differentiation of incorrectly predicted memories. *J. Neurosci.* 32 2022–2031. 10.1523/JNEUROSCI.3272-16.2017 PMC5338753 28115478

[B43] Kobayashi T. Nishijo H. Fukuda M. Bures J. Ono T. (1997). Task-dependent representations in rat hippocampal place neurons. *J. Neurophysiol.* 78 597–613. 10.1152/jn.1997.78.2.597 9307098

[B44] Kobayashi T. Tran A. H. Nishijo H. Ono T. Matsumoto G. (2003). Contribution of hippocampal place cell activity to learning and formation of goal-directed navigation in rats. *Neuroscience* 117 1025–1035. 10.1016/S0306-4522(02)00700-5 12654354

[B45] Kuhl B. A. Shah A. T. DuBrow S. Wagner A. D. (2010). Resistance to forgetting associated with hippocampus-mediated reactivation during new learning. *Nat. Neurosci.* 13 501–506. 10.1038/nn.2498 20190745 PMC2847013

[B46] Kusama T. Mabuchi M. (1970). *Stereotaxic Atlas of the Brain of Macaca fuscata: by Toshio Kusama and Masako Mabuchi.* Baltimore: University of Tokyo Press.

[B47] Lebreton M. Bertoux M. Boutet C. Lehericy S. Dubois B. Fossati P. (2013). A critical role for the hippocampus in the valuation of imagined outcomes. *PLoS Biol.* 11:e1001684. 10.1371/journal.pbio.1001684 24167442 PMC3805472

[B48] Lee H. Ghim J.-W. Kim H. Lee D. Jung M. (2012). Hippocampal neural correlates for values of experienced events. *J. Neurosci.* 32 15053–15065. 10.1523/JNEUROSCI.2806-12.2012 23100426 PMC6704836

[B49] Lisman J. E. Grace A. A. (2005). The hippocampal-VTA loop: controlling the entry of information into long-term memory. *Neuron* 46 703–713. 10.1016/j.neuron.2005.05.002 15924857

[B50] Ludvig N. Tang H. M. Gohil B. C. Botero J. M. (2004). Detecting location-specific neuronal firing rate increases in the hippocampus of freely-moving monkeys. *Brain Res.* 1014 97–109. 10.1016/j.brainres.2004.03.071 15212996

[B51] Maingret N. Girardeau G. Todorova R. Goutierre M. Zugaro M. (2016). Hippocampo-cortical coupling mediates memory consolidation during sleep. *Nat. Neurosci.* 19 959–964. 10.1038/nn.4304 27182818

[B52] Mason A. Farrell S. Howard-Jones P. Ludwig C. J. H. (2017). The role of reward and reward uncertainty in episodic memory. *J. Mem. Lang.* 96 62–77. 10.1016/j.jml.2017.05.003

[B53] Matsuda K. (1996). Measurement system of the eye positions by using oval fitting of a pupil. *Neurosci. Res.* 25 S270–S270.

[B54] Matsumura N. Nishijo H. Tamura R. Eifuku S. Endo S. Ono T. (1999). Spatial- and task-dependent neuronal responses during real and virtual translocation in the monkey hippocampal formation. *J. Neurosci.* 19 2381–2393. 10.1523/JNEUROSCI.19-06-02381.1999 10066288 PMC6782547

[B55] McNaughton B. L. Barnes C. A. O’Keefe J. (1983). The contributions of position, direction, and velocity to single unit activity in the hippocampus of freely-moving rats. *Exp. Brain Res.* 52 41–49. 10.1007/BF00237147 6628596

[B56] Miller J. F. Neufang M. Solway A. Brandt A. Trippel M. Mader I. (2013). Neural activity in human hippocampal formation reveals the spatial context of retrieved memories. *Science* 342 1111–1114. 10.1126/science.1244056 24288336 PMC4669102

[B57] Muller R. U. Kubie J. L. (1987). The effects of changes in the environment on the spatial firing of hippocampal complex-spike cells. *J. Neurosci.* 7 1951–1968. 10.1523/JNEUROSCI.07-07-01951.1987 3612226 PMC6568940

[B58] Muller R. U. Kubie J. L. Ranck J. B. (1987). Spatial firing patterns of hippocampal complex-spike cells in a fixed environment. *J. Neurosci.* 7 1935–1950. 10.1523/JNEUROSCI.07-07-01935.1987 3612225 PMC6568929

[B59] Munn R. G. Bilkey D. K. (2012). The firing rate of hippocampal CA1 place cells is modulated with a circadian period. *Hippocampus* 22 1325–1337. 10.1002/hipo.20969 21830249

[B60] Nishijo H. Ono T. Eifuku S. Tamura R. (1997). The relationship between monkey hippocampus place-related neural activity and action in space. *Neurosci. Lett.* 226 57–60. 10.1016/S0304-3940(97)00255-3 9153641

[B61] O’Keefe J. Burgess N. (1996). Geometric determinants of the place fields of hippocampal neurons. *Nature* 381 425–428. 10.1038/381425a0 8632799

[B62] O’Keefe J. Dostrovsky J. (1971). The hippocampus as a spatial map: preliminary evidence from unit activity in the freely-moving rat. *Brain Res.* 34 171–175. 10.1016/0006-8993(71)90358-1 5124915

[B63] O’Keefe J. Nadel L. (1978). *The Hippocampus as a Cognitive Map.* Oxford: Clarendon Press.

[B64] Ono T. Nakamura K. Nishijo H. Eifuku S. (1993). Monkey hippocampal neurons related to spatial and nonspatial functions. *J. Neurophysiol.* 70 1516–1529. 10.1152/jn.1993.70.4.1516 8283212

[B65] Patrono E. Matsumoto J. Nishimaru H. Takamura Y. Chinzorig I. C. Ono T. (2017). Rewarding effects of operant dry-licking behavior on neuronal firing in the nucleus accumbens core. *Front. Pharmacol.* 8:536. 10.3389/fphar.2017.00536 28860992 PMC5559468

[B66] Poucet B. Hok V. (2017). Remembering goal locations. *Curr. Opin. Behav. Sci.* 17 51–56. 10.1016/j.cobeha.2017.06.003

[B67] Quiroga R. Q. Panzeri S. (2009). Extracting information from neuronal populations: information theory and decoding approaches. *Nat. Rev. Neurosci.* 10 173–185. 10.1038/nrn2578 19229240

[B68] Quiroga R. Q. Reddy L. Koch C. Fried I. (2007). Decoding visual inputs from multiple neurons in the human temporal lobe. *J. Neurophysiol.* 98 1997–2007. 10.1152/jn.00125.2007 17671106

[B69] Rolls E. T. Xiang J.-Z. (2005). Reward-spatial view representations and learning in the primate hippocampus. *J. Neurosci.* 25 6167–6174. 10.1523/JNEUROSCI.1481-05.2005 15987946 PMC6725063

[B70] Royer S. Zemelman B. V. Losonczy A. Kim J. Chance F. Magee J. C. (2012). Control of timing, rate and bursts of hippocampal place cells by dendritic and somatic inhibition. *Nat. Neurosci.* 15 769–775. 10.1038/nn.3077 22446878 PMC4919905

[B71] Sarel A. Finkelstein A. Las L. Ulanovsky N. (2017). Vectorial representation of spatial goals in the hippocampus of bats. *Science* 355 176–180. 10.1126/science.aak9589 28082589

[B72] Scoville W. B. Milner B. (1957). Loss of recent memory after bilateral hippocampal lesions. *J. Neurol. Neurosurg. Psychiatry* 20 11–21. 10.1136/jnnp.20.1.11 13406589 PMC497229

[B73] Squire L. R. Zola-Morgan S. (1991). The medial temporal lobe memory system. *Science* 253 1380–1386. 10.1126/science.1896849 1896849

[B74] Tabuchi E. T. Mulder A. B. Wiener S. I. (2000). Position and behavioral modulation of synchronization of hippocampal and accumbens neuronal discharges in freely moving rats. *Hippocampus* 10 717–728. 10.1002/1098-1063200010:6<717::AID-HIPO1009>3.0.CO;2-3 11153717

[B75] Teles-Grilo Ruivo L. M. Baker K. L. Conway M. W. Kinsley P. J. Gilmour G. Phillips K. G. (2017). Coordinated acetylcholine release in prefrontal cortex and hippocampus is associated with arousal and reward on distinct timescales. *Cell Rep.* 18 905–917. 10.1016/j.celrep.2016.12.085 28122241 PMC5289927

[B76] Terada S. Takahashi S. Sakurai Y. (2013). Oscillatory interaction between amygdala and hippocampus coordinates behavioral modulation based on reward expectation. *Front. Behav. Neurosci.* 7:177. 10.3389/fnbeh.2013.00177 24348352 PMC3847563

[B77] Trouche S. Perestenko P. V. Ven G. M. van de Bratley C. T. McNamara C. G. Campo-Urriza N. (2016). Recoding a cocaine-place memory engram to a neutral engram in the hippocampus. *Nat. Neurosci.* 19 564–567. 10.1038/nn.4250 26900924 PMC4817230

[B78] Tryon V. L. Penner M. R. Heide S. W. King H. O. Larkin J. Mizumori S. J. Y. (2017). Hippocampal neural activity reflects the economy of choices during goal-directed navigation. *Hippocampus* 27 743–758. 10.1002/hipo.22720 28241404 PMC5479754

[B79] Tulving E. Markowitsch H. J. (1998). Episodic and declarative memory: role of the hippocampus. *Hippocampus* 8 198–204. 10.1002/(SICI)1098-106319988:3<198::AID-HIPO2>3.0.CO;2-G 9662134

[B80] Wirth S. Baraduc P. Planté A. Pinède S. Duhamel J. R. (2017). Gaze-informed, task-situated representation of space in primate hippocampus during virtual navigation. *PLoS Biol.* 15:e2001045. 10.1371/journal.pbio.2001045 28241007 PMC5328243

[B81] Wood E. R. Dudchenko P. A. Robitsek R. J. Eichenbaum H. (2000). Hippocampal neurons encode information about different types of memory episodes occurring in the same location. *Neuron* 27 623–633. 10.1016/S0896-6273(00)00071-4 11055443

[B82] Xia L. Nygard S. K. Sobczak G. G. Hourguettes N. J. Bruchas M. R. (2017). Dorsal-CA1 hippocampal neuronal ensembles encode nicotine-reward contextual associations. *Cell Rep.* 19 2143–2156. 10.1016/j.celrep.2017.05.047 28591584 PMC5524455

[B83] Zhang K. Ginzburg I. McNaughton B. L. Sejnowski T. J. (1998). Interpreting neuronal population activity by reconstruction: unified framework with application to hippocampal place cells. *J. Neurophysiol.* 79 1017–1044. 10.1152/jn.1998.79.2.1017 9463459

[B84] Zilli E. A. Hasselmo M. E. (2008). Modeling the role of working memory and episodic memory in behavioral tasks. *Hippocampus* 18 193–209. 10.1002/hipo.20382 10.1002/hipo.20382 17979198 PMC2376903

